# Case in Which, from Long-Continued Insensibility, Following Injury of the Head, It Was Deemed Necessary to Inject Fluid into the Stomach

**Published:** 1825-11

**Authors:** H. R. Oswald

**Affiliations:** Surgeon to the Household of Government, Isle of Man


ART. II.—*Case in which, from long-continued Insensibility, following Injury of the Head, it was deemed necessary to inject Fluid into the Stomach.* By H. R. OSWALD, Esq. Surgeon to the Household of Government, Isle of Man, F.A.S. &c. &c.*

MR. WM. D., aged forty, of a full but healthy and active habit of body, an auctioneer and keeper of livery stables in this town, when trying a pony at a country fair, on Friday, the 5th of August last, about noon, fell heavily backwards on his head, in consequence of the animal becoming restive whilst mounting, and thereby preventing him getting firmly into his seat, and gathering up the reins of his bridle properly. He is a sober man, and was not in any degree under the influence of liquor when he fell. He was quite stunned by the fall from the first, and immediately stretched himself on his back on the ground insensible, uttering a loud groan, to the great alarm of all who saw him. A short time after it, he was bled by a practitioner, who happened to be in the fair, but without any apparent effect whatever; and he was carried home, a distance of eight miles, the same evening, without any diminution of the insensibility.

Not being in the habit of attending Mr. D.'s family, I did not see him till the following morning, August 6th, when nothing more had been done for him than the venesection already mentioned, excepting that a large puffy tumor, situated on the occiput over the coronoid and dextroid margin of the os occipitis, had been partially laid open by the scalpel, without discovering either fracture or depression of the cranium. In fact, as the power of deglutition was destroyed, little more could be done at the first view of the case. But, as the insensibility in which I found him was of an uncommon kind, I shall premise some account of it. At short intervals, the patient appeared to sleep composedly, with half-closed eyes; and as often was very restless, changing his position, tossing his arms about the bed and over his head, and sometimes snoring with a heavy inspiration or sigh. But his breathing was not stertorous: on the contrary, it was for the most part placid, or more than usually low and easy. The eyelids were at all times partly closed, and there was a dulness and fixedness in the eye itself. The pupil contracted readily when a lighted candle was held before it; and he put up his hand to push the candle away, as if it gave him pain. He had swallowed nothing since the accident (now nineteen hours), and was not only incapable of doing so, but sometimes showed an aversion to the attempt. The power of speech was also lost, and he paid no attention to questions put to him in a loud voice. His pulse beat from 76 to 84 in the

* We shall be happy to hear from Mr. OSWALD again; and, from the interesting communication with which he has favoured us, we augur well of the other cases to which he alludes.—EDITORS.

minute, being sometimes irregular, and somewhat sharp and wiry; but, when he was roused by any effort, such as an attempt to make him swallow, or by an examination of the head, it immediately rose to 96 and 100, and upwards, according to the degree of excitement; and withal beat more feebly, and was less wiry, than when at its lowest rate. His skin was cool. He passed water freely during the night, and, when inclined to do so, felt for the pot, as a person would do in the dark. In short, he appeared much like one just upon the verge of total insensibility from intoxication, only there was more paleness than flushing of the face, and an expression of greater distress in his whole manner and appearance.

I enlarged the incision made into the tumor of the scalp, in a crucial manner, but could discover nothing like injury done to the cranium itself. I discovered, however, a livid discoloration of the integuments, between the left ear and the lower part of the mastoid process of the temporal bone, a site nearly opposite to that where the injury was received; thus indicating a tendency to that species of injury denominated counter-fracture. But there was no puffiness, or swelling, or pain on pressure in the discoloured place; and, therefore, I did not consider myself warranted in proposing to lay the integuments open, for the purpose of ascertaining the nature of the injury done here: and this opinion gained strength by a consideration of the structure of the parts adjacent and interior to the mastoid process.

There being a disagreement in opinion betwixt the two medical gentlemen in attendance before me, respecting the further detraction of blood, the one fearing collapse, the other congestion and effusion in the brain; and as it was also mentioned that blood could not be obtained by venesection, owing to the depth and contracted state of the veins, I advised the immediate application of three dozen of leeches to the occiput. The discharge of blood begun by them was promoted for several hours, and was very considerable. The domestic glyster, and other enemata, with infusion of senna and salts, and solutions of the tartarised antimony, to the extent of ten grains and a scruple, were successively administered during the course of the day, before the bowels were freely evacuated. This practice was productive of some relief to the restlessness, and was also followed by some improvement in the pulse; but the insensibility, as above described, continued unabated.

11 P.M. 6th August.—Little change is perceptible in the case. Mouth, and teeth, and tongue are parched, and partially furred with black sordes; and the eyes are, if any thing, more inanimate.—Let a blister be applied to the nape of the neck, and an opiate be administered by enema to-night.

August 7th, 11 A.M.—Still insensible as before. Night reported to have been good,—that is, quiet. Pulse eighty, somewhat compressible, but without irregularity; still becomes more frequent upon any exertion. Has swallowed nothing, and liquids poured into his mouth run out again without irritating the fauces, as if he was unconscious of them; sometimes shuts his mouth against the spoon. Two injections of diluting and pabulous fluids were thrown up during the course of the night and morning.—Repeat the leeches again to the extent of three dozen, and the purgative enemata.

4 P.M.—No change, except that, when asked to show his tongue, seems to make an obscure attempt to do so. Glysters have operated, and blister has risen well. Heat of surface natural, but there is more tendency to a cool state of the skin than to heat. Temperature of the head is, perhaps, somewhat greater than that of the body. Mouth black and parched, but he shows no symptoms of thirst, although he has been upwards of fifty hours without swallowing any thing, and has frequently evinced a desire to pass urine. The leech-bites continue discharging: the warm poultices applied to them, to encourage the discharge from them, give manifest relief to the uneasiness. It has also been observed that he is more composed when the leeches are in the act of fixing on and filling. Can this relief be of an electrical nature? I have often thought that leeches gave more relief than the quantity of blood evacuated by them could lead us to expect.—Repeat the nutritive and diluting injections, and apply cold lotions to the head.

I had proposed, at my morning-visit, the possibility of throwing some pabulous fluid, as well as medicine, into the stomach, by means of a simple syringe with a flexible tube, which I constructed myself six years ago, with the assistance of a saddler in this town. By this means I expected to obviate some of the bad effects proceeding from the loss of the power of deglutition,—such as the want of nourishment, and the torpor in which the superior portions of the alimentary canal must be, from the absence of all stimuli of a diluting, pabulous, or medicated kind.

No practitioner in the island was in possession of the superior instruments of Jukes or of Read;* and, as for myself, I had hitherto remained satisfied with possessing my own simple one. I had constructed this for the purpose of pumping water from the stomach in cases of drowning, the treatment of which, to a practitioner situated in a sea-port town, is not unfrequently a most painful duty: for I had observed that no patient is taken completely insensible out of the water, in which the stomach is not loaded with a large quantity of water, and that this water causes frequent retchings, and often severe vomiting, during resuscitation, and is always discharged, either upwards or downwards, before recovery is complete; and therefore concluded that the pressure of this large quantity of water upon the diaphragm, and the chilling effect it necessarily had upon the stomach itself, retarded, in a considerable degree, the resuscitation in unsuccessful cases. This inference may be erroneous,

* This defect has since been supplied.

and I do not mean to argue it, but only to state that under the impression of it I constructed my syringe, which most likely would never have been named to the public, had it not been for the case at present under detail: I therefore beg you will excuse this digression.

7 P.M.—Coma appears still more profound than in the forenoon, and the pulse is still more easily compressible; and other symptoms are such as to offer no hopes of recovery, but, on the contrary, to increase our fear of collapse. It was therefore agreed that we were warranted in introducing a pint of fluid, consisting of equal parts of skim-milk and common tea, into the stomach, by means of the instrument just mentioned. This was a metal syringe, capable of holding somewhat more than half a pint, fitted by means of a screw to a flexible tube, formed of spiral wire covered with leather, twenty inches long, and terminated by a smooth piece of bone perforated, but having neither valve nor stop-cock to regulate its operation. With the assistance of Mr. Hector and myself, Mr. Costain introduced the tube into the œsophagus, after the usual manner. The patient was roused to considerable resistance to its introduction into the stomach; for, making a loud inarticulate noise, he suddenly got hold of it on the first attempt, and pulled it forcibly back again. The forcible attempts he made to get his hands loose after they were held, the loud efforts of his voice, and the impediment to respiration which the pressure of the tube on the larynx occasioned, gave rise to a suffusion and turgidity of the countenance, which almost staggered our conviction that the practice was justifiable; therefore, having quickly injected a pint of tea and milk, the instrument was laid aside. Immediately after the operation, the pulse was 125, small, and somewhat fluttering, (if I may use the expression;) and the patient relapsed directly into his former state of quiescent stupor and insensibility.

11 P.M.—Pulse has subsided to eighty, and is regular; skin is soft; and he has slept soundly, during longer periods, than before the operation.—Let the diluent enema be repeated directly, with the addition of twenty drops of Træ. opii, to prevent the immediate return of it.

8th August.—To our great satisfaction, our patient has regained, in some degree, the powers of speech, of hearing, and of swallowing. This change was first perceived at six o'clock this morning, when he inquired what o'clock it was? Says he is "very ill," and repeats the same thing often in a childish manner. Has little else to say, except what his wife calls childish peevish questions. Only swallows a tea-spoonful or two at a time, and that slowly. Complains of oppressive headache, and refers the pain principally to the top of the head; the temperature of which feels preternaturally increased. Does not feel the wounded part in the smallest degree, and says he has only a little pain in the occiput and base of the cranium. Pulse eighty-two, and regular.—Repeat three dozen of leeches to the head; apply a blister to the scalp, and a cold lotion before the blister is sent; and let him have the following powder directly:—℞. Pulveris Jalapæ, ℈ss.; Hydrargyri Submuriatis, gr. vj. M.

7 P.M.—Facility of deglutition returns slowly. Medicine has operated well, and the leeches have been applied: but he complains still more of headache, and the temperature of the head is also increased. Pulse ninety-four, and harder than it has yet been. Suspecting the commencement of the inflammatory stage of concussion of the brain, so well distinguished from the first and second stages by Mr. Abernethy, I immediately opened a vein in the arm myself; and, after a pint had been taken, the blood ceasing to flow freely, I tied the other arm up also, and took twenty ounces more in a full stream, when his headache became easier, and the pulse almost disappeared at the wrist; but there was hardly any other symptom of approaching syncope. However, the patient, having occasion to get up about half an hour after, fainted on the floor.

11 P.M.—Headache much relieved, and the manner and general expression of the patient improved. Pulse ninety, soft and compressible; but he complains of restlessness, which is most probably owing to the loss of blood he has sustained.—Give him diluents liberally, and repeat the anodyne enema at night.

9th August.—Has passed a tolerable night. Headache much better; but still complains, in a childish manner, of being "very ill." Wishes for something solid to eat,—beef-steaks for example,—with beer to drink. Has taken a good deal of fluids during the night. Deglutition is now little impeded.—Let him take the following infusion directly: ℞. Infusi Sennæ, ℥iv. Sulphatis Magnesiæ, ʒvj. solve; and continue his regimen of water-gruel and tea.

10th August.—Continues to improve; headache little felt.—Continuantur omnia.

11th.—Convalescence. Continues to solicit solid food; and, in consequence of a still more earnest desire for beer, was allowed to have a little ginger-beer to-day. Wound and injury of the scalp quite healed, having closed by the first intention.

12th.—Instead of half a tumbler, drank three bottles of ginger-beer yesterday. Complains more of his head to-day, and is restless, and his skin is heated and dry.—Omit the ginger-beer, and let him take directly ℞. Extracti Colocynth. compos. gr. x. Hydrargyri Submuriatis gr. vj. in forma pilularum; et rep. Enema si opus sit.

13th.—Is better again.

From this period he continued to recover rapidly; and in a few days insisted upon taking a drive in a close carriage, with a degree of urgency almost amounting to delirium, but which was indicated by no other symptoms, excepting, perhaps, a slight increase of the temperature of the head. He was indulged in his fancy, because resisting it gave rise to a greater degree of irritation than the drive could produce. From the same kind of imbecility of mind, he was permitted to go through two days' work as an auctioneer, not much exceeding a fortnight after his accident. This was an engagement he had entered into previously, and which incessantly occupied his mind from the time that his recollection returned.

27th.—Continues free of complaint, excepting an unusual degree of lassitude, and occasionally a sensation of giddiness, and a painful sensation in the occiput.

As some information respecting the nature of the affection may be elicited by his own recollections of what took place during his illness, I shall state them here. He remembers being in the fair; but recollects nothing about the pony, about mounting it, or falling from it; recollects nothing till the Monday following the accident, and even on that day only a few things: as an example, he instances having recognised me during the morning visit. Being questioned relative to the operation, thinks he remembers indistinctly having suffered an impression of pain about the throat, but nothing more.

His having no recollection of the pony, or of having mounted it, has a tendency to lead us to infer that some apoplectic congestion existed previous to the injury; and this is in some degree his own suspicion: but the marks of external injury, especially the discoloration of the integuments behind the left ear, are sufficient evidence of the nature of the case. But it is unnecessary for me to remark upon it,—it is sufficiently long already. I may just observe, however, that I hold it to be a case of unequivocal concussion of the brain. The patient received a knock-down or stunning blow, so severe that it caused blackness on the opposite side of the head, from which he did not recover immediately, as is usually the case in slight concussions, but remained sixty-five hours insensible; and would probably never have recovered, had not injection into the stomach been practised, so as to rouse re-action in that organ, and consequently in the whole system. Very common case.

